# Molecular characterization of SARS-CoV-2 detected in Tokyo, Japan during five waves: Identification of the amino acid substitutions associated with transmissibility and severity

**DOI:** 10.3389/fmicb.2022.912061

**Published:** 2022-07-27

**Authors:** Koji Tsuchiya, Norio Yamamoto, Yoshie Hosaka, Mitsuru Wakita, Makoto Hiki, Yasushi Matsushita, Hirotake Mori, Satoshi Hori, Shigeki Misawa, Takashi Miida, Shuko Nojiri, Kazuhisa Takahashi, Toshio Naito, Yoko Tabe

**Affiliations:** ^1^Department of Clinical Laboratory, Juntendo University Hospital, Bunkyo, Tokyo, Japan; ^2^Department of General Medicine, Juntendo University Graduate School of Medicine, Bunkyo-ku, Japan; ^3^Department of Microbiology, Juntendo University Graduate School of Medicine, Bunkyo-ku, Japan; ^4^Department of Microbiology, Tokai University School of Medicine, Isehara, Japan; ^5^Department of Emergency Medicine, Juntendo University Faculty of Medicine, Bunkyo-ku, Japan; ^6^Department of Cardiovascular Biology and Medicine, Juntendo University Graduate School of Medicine, Bunkyo-ku, Japan; ^7^Department of Internal Medicine and Rheumatology, Juntendo University Graduate School of Medicine, Bunkyo-ku, Japan; ^8^Department of Respiratory Medicine, Juntendo University Graduate School of Medicine, Bunkyo-ku, Japan; ^9^Department of Infection Control Science, Juntendo University Graduate School of Medicine, Bunkyo-ku, Japan; ^10^Department of Clinical Laboratory Medicine, Juntendo University Faculty of Medicine, Bunkyo-ku, Japan; ^11^Medical Technology Innovation Center, Juntendo University, Bunkyo-ku, Japan; ^12^Department of Research Support Utilizing Bioresource Bank, Juntendo University Graduate School of Medicine, Bunkyo-ku, Japan

**Keywords:** SARS-CoV-2, COVID-19, Japan, genome sequencing, transmissibility, severity

## Abstract

Many variants of SARS-CoV-2 have emerged around the world. It is therefore important to understand its global viral evolution and the corresponding mutations associated with transmissibility and severity. In this study, we analyzed 112 whole genome sequences of SARS-CoV-2 collected from patients at Juntendo University Hospital in Tokyo and the genome data from entire Japan deposited in Global Initiative on Sharing Avian Influenza Data (GISAID) to examine the relationship of amino acid changes with the transmissibility and the severity of each strain/lineage. We identified 12 lineages, including B.1.1.284, B.1.1.214, R.1, AY.29, and AY.29.1, which were prevalent specifically in Japan. B.1.1.284 was most frequently detected in the second wave, but B.1.1.214 became the predominant lineage in the third wave, indicating that B.1.1.214 has a higher transmissibility than B.1.1.284. The most prevalent lineage during the fourth and fifth wave was B.1.1.7 and AY.29, respectively. In regard to the severity of identified lineages, B.1.1.214 was significantly lower than the reference lineage, B.1.1.284. Analysis of the genome sequence and other traits of each lineage/strain revealed the mutations in S, N, and NSPs that increase the transmissibility and/or severity. These mutations include S: M153T, N: P151L, NSP3: S543P, NSP5: P108S, and NSP12: A423V in B.1.1.284; S: W152L and E484K in R.1; S: H69del, V70del, and N501Y in the Alpha strain; S: L452R, T478K, and P681R in the Delta strain. Furthermore, it is suggested that the transmissibility of B.1.1.214 could be enhanced by the mutations N: M234I, NSP14: P43L, and NSP16: R287I. To address the issue of the virus evolution, it is necessary to continuously monitor the genomes of SARS-CoV-2 and analyze the effects of mutations for developing vaccines and antiviral drugs effective against SARS-CoV-2 variants.

## Introduction

In December 2019, several cases of unknown pneumonia were detected in the city of Wuhan, Hubei province, China. Deep sequencing identified the causative agent as a novel coronavirus, which was named nCoV-2019 and later renamed to SARS-CoV-2 (Coronaviridae Study Group of the International Committee on Taxonomy of Viruses, 2020; Huang et al., 2020; Wu et al., 2020; Zhu et al., 2020).

Rapidly spreading throughout the world, the World Health Organization (WHO) officially declared the SARS-CoV-2 outbreak as a pandemic on March 11, 2020 (World Health Organization, 2020). As of March 31, 2022, there have been 485,369,784 confirmed cases and 6,138,368 deaths around the world.^1^ In Japan, the first domestic case was identified on January 16, 2020, and, after five waves, a total of 1,716,928 positive cases were detected by the end of October 2021.^2^

The SARS-CoV-2 genome encodes 14 open reading frames: Orf1a/ab, four structural proteins (S, E, M, and N), and nine putative accessory proteins (Hoque et al., 2020; Islam et al., 2020; Rahman et al., 2020, 2021b). The Orf1a/ab is a large polyprotein and is proteolytically processed into 16 non-structural proteins (NSPs; Hoque et al., 2020). One of them, NSP14, consists of an N-terminal exonuclease domain and a C-terminal N7-MTase domain (Ogando et al., 2020). NSP14 functions as a proofreading molecule that reduces the error rate during replication. Although SARS-CoV-2 does not show as high of a mutation rate as other RNA viruses, many SARS-CoV-2 variants are emerging because of the huge amount of viral replication from the large number of infected hosts. As a result of extended human-to-human transmission, SARS-CoV-2 has obtained amino acid changes with fitness advantages. It was reported that D614G (Korber et al., 2020; Yurkovetskiy et al., 2020; Volz et al., 2021a) and N501Y (Liu et al., 2022) in S protein enhance the transmissibility of SARS-CoV-2. In addition to S protein mutations, nucleocapsid mutations R203K and G204R were found to increase the infectivity, fitness, and pathogenicity of SARS-CoV-2 (Rahman et al., 2021a; Wu et al., 2021). The amino acid substitution P323L in NSP12 (RNA-dependent RNA polymerase) was also identified as the highly prevalent mutation, and the significant association between the presence of P323L and severe disease was reported (Flores-Alanis et al., 2021). The deletions of the viral genes such as 382-nt deletion of ORF8 (Young et al., 2020), 81-nt deletion of ORF7a (Holland et al., 2020), 30-nt deletion of spike protein (Lau et al., 2020), and 24-nt deletion of NSP1 (Islam et al., 2020) were found, which were predicted to influence the viral adaptation or attenuation by affecting the structures and functions of the proteins (Hoque et al., 2020; Islam et al., 2020).

Since the continuous replication of SARS-CoV-2 leads to the emergence and spread of new variants with higher transmissibility and varying severity, it is necessary to monitor all of the local diversity of SARS-CoV-2 variants to understand its global viral evolution and the association of mutations with transmissibility and severity.

In this study, we performed a comprehensive genomic analysis of 112 SARS-CoV-2 strains detected at Juntendo University Hospital in Tokyo and an analysis of the sequence data from entire Japan deposited in Global Initiative on Sharing Avian Influenza Data (GISAID) to understand the changing trend of SARS-CoV-2 genomes and find the correlation of amino acid changes with the transmissibility and severity of each lineage. Investigation of the mutations in viral genomes and analysis of their phenotype will be necessary for developing effective vaccines and antivirals against variants of SARS-CoV-2 (Hoque et al., 2020; Islam et al., 2020).

## Materials and methods

### Specimen collection and testing

Clinical samples were collected between March 1, 2020, and July 31, 2021, at Juntendo University Hospital. One hundred and twelve specimens where SARS-CoV-2 N gene were detected with less than 30 cycle threshold (Ct) values by real-time RT-PCR tests were utilized for this study following the WHO recommendation that specimens tested positive for COVID-19 with Ct value <30 are considered good materials for sequencing the whole genome of SARS-CoV-2 (Operational considerations for COVID-19 surveillance using GISRS: interim guidance, 26 March 2020; World Health Organization, 2020). Frozen-stored nasopharyngeal swab specimens in phosphate-buffered saline (PBS) and saliva samples (−80°C, single freeze–thaw) from patients with COVID-19 were used.

### Whole genome sequencing of SARS-CoV-2

One hundred and twelve purified RNAs were reverse-transcribed into cDNAs using the SuperScript VILO cDNA synthesis kit (Invitrogen, Carlsbad, CA, United States). The synthesized cDNAs were amplified with the Ion AmpliSeq SARS-CoV-2 Research Panel (Thermo Fisher Scientific, Waltham, MA, United States) in the Ion GeneStudio S5 System according to the manufacturer’s instructions. The Ion AmpliSeq SARS-CoV-2 Research Panel consists of two primer pools targeting 237 amplicons tiled across the SARS-CoV-2 genome, with an additional five primer pairs targeting human expression controls. The SARS-CoV-2 amplicons range from 125 to 275 bp in length. Amplified samples were then sequenced using Ion 530 chips (Thermo Fisher Scientific) with eight samples per chip on the Ion S5 system. The Torrent Suite 5.14.0 platform and specific plugins were used for Next-Generation Sequencing (NGS) data analysis. All analyzed sequences showed an alignment accuracy of over 96% and a base coverage over 50×. The pangolin software was used for the assignment of SARS-CoV-2 lineages. All sequences were then submitted as FASTA files and deposited in the EpiCoV database of GISAID (Shu and McCauley, 2017). The accession numbers of these sequences were shown in Supplementary Table 1. Amino acid substitutions in the sequenced viruses were analyzed with GISAID during the registration of the viral genomes, and the information was collected from the EpiCoV database. Analysis of PANGO lineage was performed based on v.3.1.15. Moreover, we analyzed the genome data deposited in GISAID (97,458 complete sequences collected from March 1, 2020 to July 31, 2021 in Japan) to compare the patterns of the prevalent lineages between the samples collected at Juntendo University Hospital and those throughout Japan.

### Phylogenetic tree analysis

A total of 121 nucleotide sequences (112 sequences from Juntendo University Hospital and nine reference sequences) were aligned with the MUSCLE program. There were a total of 29,906 positions in the final dataset. The evolutionary history was inferred by using the Maximum Likelihood method and General Time Reversible model in MEGA 11. Initial tree for the heuristic search was obtained automatically by applying Neighbor-Join and BioNJ algorithms to a matrix of pairwise distances estimated using the Maximum Composite Likelihood (MCL) approach. A discrete Gamma distribution was used to model evolutionary rate differences among sites [five categories (+G, parameter = 0.1000)]. The rate variation model allowed for some sites to be evolutionarily invariable ([+I], 48.99% sites). The tree with the highest log likelihood (−47424.62) was selected for presentation.

### The severity of COVID-19 patients at Juntendo University Hospital

The severity of COVID-19 was categorized into four levels according to the WHO criteria (World Health Organization, 2021). Briefly, the mild type was defined as patients with mild clinical symptoms, but no evidence of viral pneumonia or hypoxia. The moderate type was defined as patients with fever, respiratory symptoms, or other symptoms, but with no evidence of severe pneumonia, including SpO_2_ ≥ 90% on room air. The severe type was defined as patients with clinical signs of pneumonia and at least one of the following: shortness of breath (breathing rate ≥ 30/min), SpO_2_ < 90% on room air, or severe respiratory distress. The critical type was defined as patients with any of the following symptoms: respiratory failure requiring mechanical ventilation, shock, or a combination of other organ failures requiring ICU monitoring treatment.

### Statistical analysis

We collected the information about the infected patients including the severity and the lineage data of SARS-CoV-2 determined by whole genome sequencing. To analyze the factors associated with the severity, we re-categorized the severity status into the two groups. The mild and moderate were defined as the less severe group, and the severe and critical are defined as the severe group. We constructed unadjusted and adjusted logistic regression models for the re-defined severity risk, adjusted the relevant factors (i.e., age and sex), and estimated the effect of the lineage with B.1.1.284 as the reference. Odds Ratios (ORs) and 95% CIs were estimated. A two-sided *α* of < 0.05 was considered statistically significant. Statistical analyses were conducted using SAS version 9.4 (SAS Institute, Cary, North Carolina).

### Ethical approval

This study complied with all relevant national regulations and institutional policies and was conducted in accordance with the tenets of the Helsinki Declaration. This study was approved by the institutional review board (IRB) at Juntendo University Hospital (IRB #20–036). The need for informed consents from individual patients was waived because all samples were de-identified in line with the Declaration of Helsinki.

## Results

### Analysis of SARS-CoV-2 lineages in the patients at Juntendo University Hospital in Tokyo, Japan

**Figure 1:**
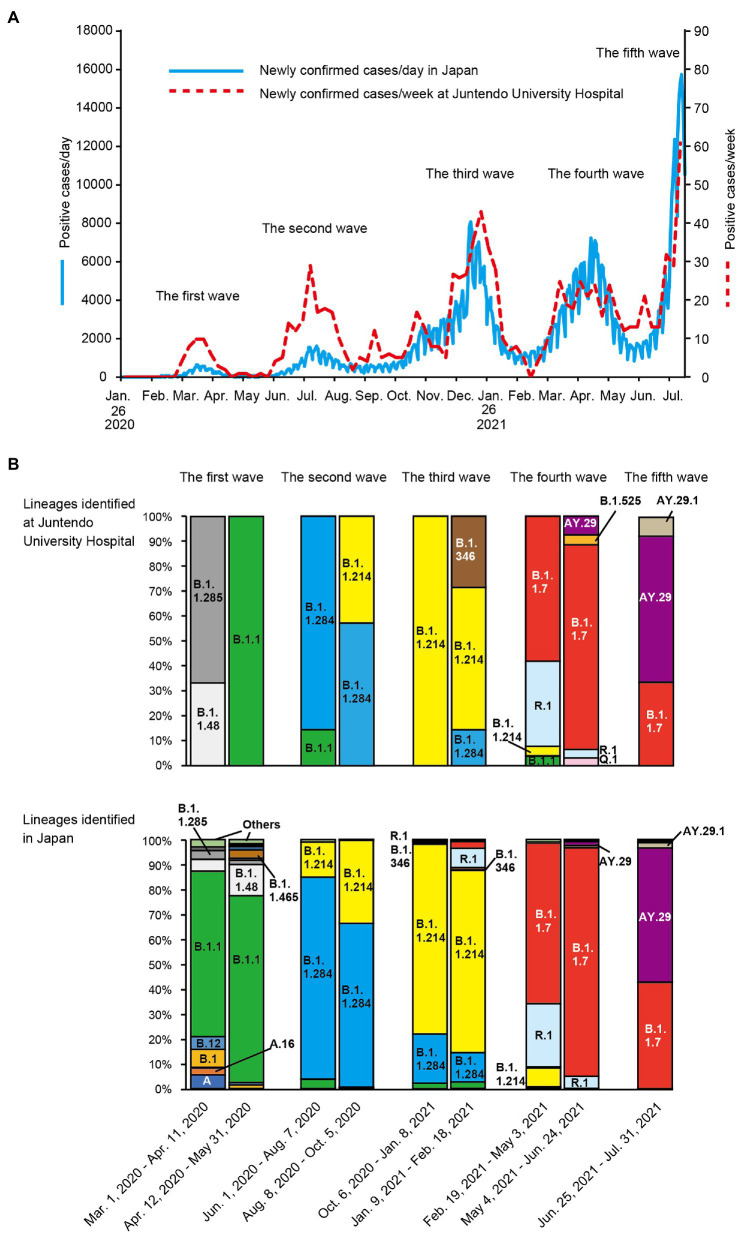
Trend of SARS-CoV-2 positive cases and lineage analysis of the viruses in each wave. **(A)** the numbers of newly confirmed cases per week in Japan and newly confirmed cases per day at Juntendo University Hospital are shown by the blue solid line and the red dotted line, respectively. **(B)** the ratios of the identified lineages at Juntendo University Hospital and in entire Japan during the designated periods are displayed.

To determine the genetic characteristics of the SARS-CoV-2 detected at Juntendo University Hospital, we performed whole genome sequencing of clinical specimens. Throughout the five waves, a total of 970 cases were identified as positive for SARS-CoV-2 *via* RT-PCR at Juntendo University Hospital (Figure 1A). One hundred and twelve specimens with a lower Ct value were selected for sequencing analysis. We identified 12 lineages, such as B.1.1, B.1.1.284, B.1.1.214, and AY.29 (Supplementary Table 1). During the first wave, B.1.1 was most frequently detected, but the predominant lineage became B.1.1.284 in the second wave (Figure 1B). The most prevalent lineage during the third, fourth, and fifth wave was B.1.1.214, B.1.1.7 (the Alpha strain), and AY.29 (the Delta strain), respectively. In the fourth wave, R.1 was the second most frequent lineage. AY.29.1, a sub-lineage of AY.29, was also found in the fifth wave. Furthermore, we compared our data with the genome sequences of prevalent viruses in Japan that were downloaded from GISAID (97,458 sequences). We found that the patterns of the dominant lineages during five waves were similar between our data and the data of Japan (Figure 1B).

### Phylogenetic analysis of SARS-CoV-2 genomes identified at Juntendo University Hospital

**Figure 2:**
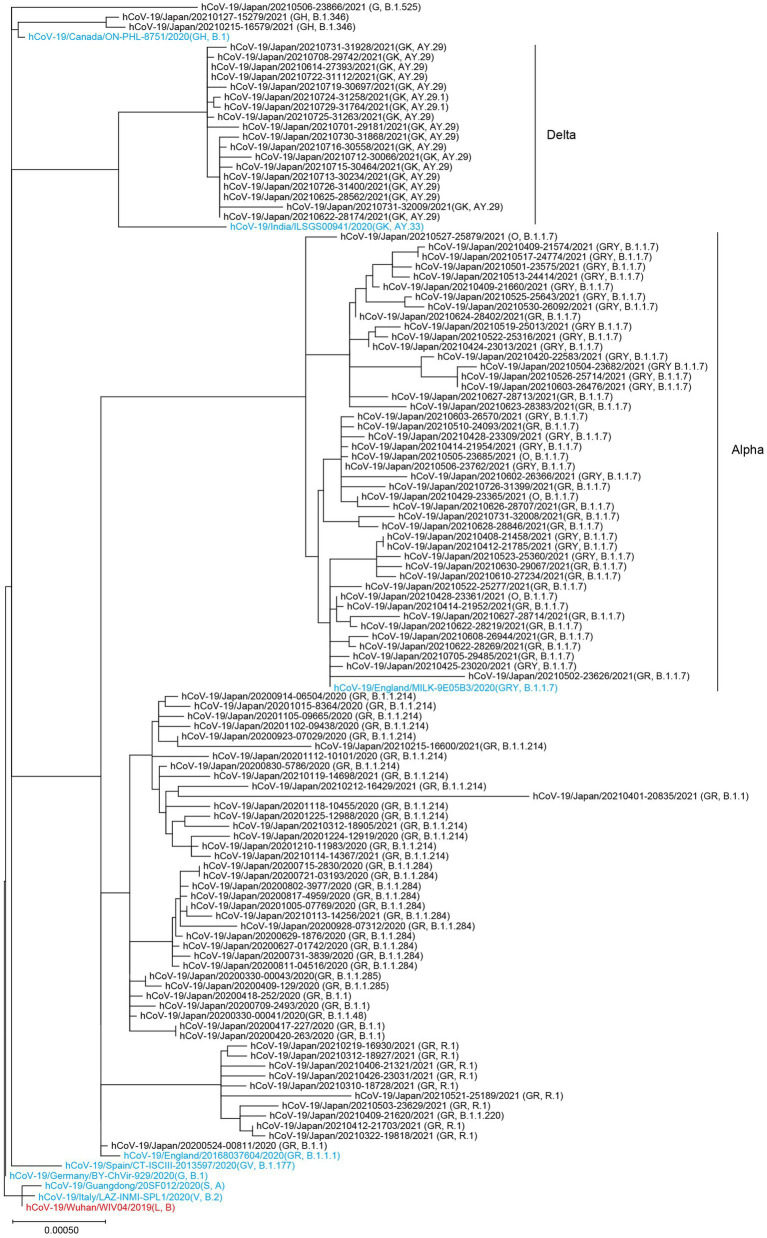
Phylogenetic tree of the SARS-CoV-2 genomes collected at Juntendo University Hospital. This tree includes 112 genomes from Juntendo University Hospital and nine reference sequences. The reference strain hCoV-19/Wuhan/WIV04/2019 is shown in red and the other references are shown in blue.

The SARS-CoV-2 genomes sequenced at Juntendo University Hospital formed three clusters consisting of the GISAID clades GR/GRY/O, GH, and GK (Figure 2). The GR/GRY/O cluster was composed of four subclusters, each of which included B.1.1.284, B.1.1.214, R.1, and the Alpha strain, respectively. B.1.1.284 and B.1.1.214 were the domestic lineages that circulated mainly in Japan, while R.1 was chiefly identified in the United States and Japan. The subcluster of the Alpha strain included B.1.1.7 and Q.1. The two viruses belonging to the B.1.346 lineage were situated in the GH clade, with the reference strain derived from Canada. The GK cluster contained the lineages AY.29 and AY.29.1, both of which were the Delta strain chiefly identified in Japan.

### Analysis of the amino acid changes in SARS-CoV-2 genomes detected at Juntendo University Hospital

**Figure 3:**
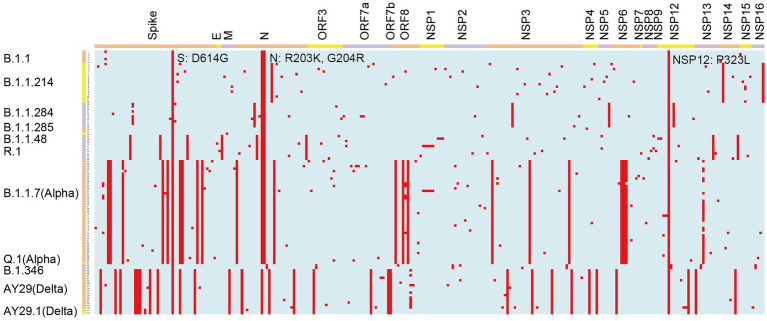
Graphical representation of amino acid changes in the SARS-CoV-2 genomes identified at Juntendo University Hospital. Amino acid substitutions are colored in red. The viral genes are shown on the top and the lineages are indicated on the left.

We analyzed 112 SARS-CoV-2 genomes and identified 2,209 amino acid changes (Table 1; Figure 3). Of these amino acid changes, 736 mutations were found in the S protein, which plays a vital role in SARS-CoV-2 infection. The most common change in the S region was D614G. We observed 395 amino acid changes in the N protein, where R203K and G204R were the most widespread mutations. In addition to structural proteins, such as S and N, there were 786 amino acid alterations in ORF1ab, which were cleaved into 16 nonstructural proteins (NSPs). All of the analyzed viruses possessed the amino acid substitution P323L in NSP12.

**Table 1:** The number of amino acid changes observed in SARS-CoV-2 sequenced at Juntendo University Hospital.

Genome segment		Missense mutation	In-frame deletion	Stop-gained	Total
Spike		574	162	0	736
E		2	0	0	2
M		33	0	0	33
N		395	0	0	395
ORF3		35	0	0	35
ORF7a		54	1	0	55
ORF7b		18	0	0	18
ORF8		107	0	42	149
ORF1a/ab	NSP1	2	13	0	15
	NSP2	20	0	0	20
	NSP3	243	0	0	243
	NSP4	41	0	0	41
	NSP5	15	0	0	15
	NSP6	26	126	0	152
	NSP7	1	0	0	1
	NSP8	4	0	0	4
	NSP9	3	0	0	3
	NSP12	149	0	0	149
	NSP13	64	0	0	64
	NSP14	51	0	0	51
	NSP15	7	0	0	7
	NSP16	21	0	0	21
Total		1,865	302	42	2,209

In B.1.1, which was the most frequently detected lineage during the latter period of the first wave, four amino acid substitutions were commonly identified: S: D614G, N: R203K, N: G204R, and NSP12: P323L (Table 2; Figure 3). These mutations have been maintained in many other lineages. B.1.1.284, the most predominant lineage in the former and latter period of the second wave, had S: M153T and N: P151L in the structural proteins, and NSP3: S543P, NSP5: P108S, and NSP12: A423V in the non-structural proteins in addition to the common mutations. Regarding B.1.1.214, the major lineage before and after the peak of the third wave, no amino acid substitutions were found in the spike region except for D614G. In the other region of this lineage, only N: M234I, NSP14: P43L, and NSP16: R287I were observed. The Alpha viruses, including B.1.1.7 and Q.1 (the most widespread during the former and latter period of the fourth wave), had 25 amino acid changes: 10 changes in S; four changes in N; three changes in ORF8, NSP3, and NSP6; and one change in NSP12 and NSP13. Mutations in S protein involved H69del, V70del, Y144del, N501Y, A570D, D614G, P681H, T716I, S982A, and D1118H. The second most prevalent lineage in the former period of the fourth wave was R.1, where 12 mutations were identified, including S: W152L, S: E484K, S: D614G, and S: G769V. The Delta viruses (AY.29 and AY.29.1) were heavily mutated and had 32 amino acid changes: 11 changes in Spike; four changes in N and NSP3; two changes in ORF7a, NSP4, and NSP12; and one change in M, ORF3, ORF7b, ORF8, NSP6, NSP13, and NSP14. S protein mutations were as follows: T19R, T95I, G142D, E156G, F157del, R158del, L452R, T478K, D614G, P681R, and D950N.

**Table 2:** Amino acid substitutions observed in the representative lineage for each wave in comparison with hCoV-19/Wuhan/WIV04/2019.

Lineage	B.1.1	B.1.1.284	B.1.1.214	R.1	Alpha	Delta
Period	1st wave	2nd wave	3rd wave	4th wave	4th wave	5th wave
Amino acid changes	S: L54F	S: M153T	S: D614G	S: W152L	S: H69del	S: T19R
	S: D614G	S: D614G		S: E484K	S: V70del	S: T95I
				S: D614G	S: Y144del	S: G142D
				S: G769V	S: N501Y	S: E156G
					S: A570D	S: F157del
					S: D614G	S: R158del
					S: P681H	S: L452R
					S: T716I	S: T478K
					S: S982A	S: D614G
					S: D1118H	S: P681R
						S: D950N
				M: F28L		M: I82T
	N: R203K	N: P151L	N: R203K	N: S187L	N: D3L	N: D63G
	N: G204R	N: R203K	N: G204R	N: R203K	N: R203K	N: R203M
		N: G204R	N: M234I	N: G204R	N: G204R	N: G215C
				N: Q418H	N: S235F	N: D377Y
					ORF8: Q27stop	ORF3: S26L
					ORF8: R52I	ORF7a: V82A
					ORF8: Y73C	ORF7a: T120I
						ORF7b: T40I
						ORF8: P93S
	NSP12: P323L	NSP3: S543P	NSP12: P323L	NSP12: P323L	NSP3: T183I	NSP3: A488S
		NSP5: P108S	NSP14: P43L	NSP13: G439R	NSP3: A890D	NSP3: V932A
		NSP12: P323L	NSP16: R287I	NSP14: P412H	NSP3: I1412T	NSP3: P1228L
		NSP12: A423V			NSP6: S106del	NSP3: P1469S
					NSP6: G107del	NSP4: V167L
					NSP6: F108del	NSP4: T492I
					NSP12: P323L	NSP6: T77A
					NSP13: E261D	NSP12: P323L
						NSP12: G671S
						NSP13: P77L
						NSP14: A394V

### Severity of the patients’ symptoms who were infected with the representative lineages at Juntendo University Hospital

To determine whether some lineages had different pathogenicity from the others, the severity of COVID-19 patients’ symptoms in Juntendo University Hospital was evaluated. B.1.1.284 was used as the basis for comparison because B.1.1.284 was the earliest lineage that was included in more than 10 samples in our study. We found that the severity of the patients with B.1.1.214 was significantly lower than those with B.1.1.284 as shown in Tables 3, 4 (odds ratio 0.08, 95%CI 0.01–0.84, *p* = 0.0277 in the univariate model; odds ratio 0.04, 95%CI 0.00–0.58, *p* = 0.0373 in the multivariate model). B.1.1, R.1, Alpha, and Delta exhibited no significant difference in the severity in comparison with B.1.1.284. Since the patients infected with the Delta strain involved vaccinated and unvaccinated individuals (11 unvaccinated; six vaccinated once; and one vaccinated twice), we also compared the severity between those with and without vaccination. There was no significant difference in the severity between vaccinated and unvaccinated Delta-infected patients in this study (*p* = 0.5842 in the univariate model; *p* = 0.1571 in the multivariate model).

**Table 3:** Severity of the patients infected with the representative lineages at Juntendo University Hospital.

Severity	The number of patients (% of total)							
	B.1.1	B.1.1.284	B.1.1.214	R.1	Alpha (B.1.1.7 + Q.1)	Delta (AY.29 + AY.29.1)	Others	All lineages
Mild	3 (50.0%)	4 (36.4%)	12 (75.0%)	3 (30.0%)	12 (26.7%)	7 (38.9%)	1 (16.7%)	42 (37.5%)
Moderate	1 (16.7%)	2 (18.2%)	3 (18.8%)	2 (20.0%)	12 (26.7%)	4 (22.2%)	3 (50.0%)	27 (24.1%)
Severe	2 (33.3%)	3 (27.3%)	1 (6.3%)	5 (50.0%)	19 (42.2%)	7 (38.9%)	2 (33.3%)	39 (34.8%)
Critical	0 (0.0%)	2 (18.2%)	0 (0.0%)	0 (0.0%)	2 (4.4%)	0 (0.0%)	0 (0.0%)	4 (3.6%)
Total	6 (100.0%)	11 (100.0%)	16 (100.0%)	10 (100.0%)	45 (100.0%)	18 (100.0%)	6 (100.0%)	112 (100.0%)

**Table 4:** Association between genotype and risks of severity in COVID-19 patients.

Variables	Univariate			Multivariate^*^		
	Odds ratio (95% CI)		*p* value	Odds ratio (95% CI)		*p* value
**Age**	**1.07**	**(1.04–1.10)**	**< 0.0001**	**1.09**	**(1.05–1.13)**	**<0.0001**
Sex	2.24	(0.97–5.15)	0.0584	2.25	(0.76–6.64)	0.1409
Vaccine	0.62	(0.12–3.37)	0.5842	0.18	(0.02–1.95)	0.1571
Lineage						
Alpha vs. B.1.1.284	1.05	(0.28–3.94)	0.1315	0.80	(0.13–4.98)	0.0303
B.1.1 vs. B.1.1.284	0.60	(0.08–4.76)	0.9986	0.22	(0.02–3.10)	0.7775
**B.1.1.214 vs. B.1.1.284**	**0.08**	**(0.01–0.84)**	**0.0277**	**0.03**	**(0.00–0.56)**	**0.0299**
Delta vs. B.1.1.284	0.76	(0.17–3.49)	0.6216	0.98	(0.11–8.88)	0.0851
R.1 vs. B.1.1.284	1.20	(0.22–6.68)	0.9986	0.07	(0.00–1.49)	0.2066
Others vs. B.1.1.284	0.60	(0.08–4.76)	0.2472	0.34	(0.03–3.75)	0.8152

* ORs and 95% CIs were estimated by logistic regression model adjusting for age and sex. The bold values mean that the difference was significant (p value was less than 0.05 and 95% CI did not cross 1).

Furthermore, we observed that age was significantly associated with the progression of symptoms (odds ratio 1.07, 95%CI 1.04–1.10, *p* < 0.0001 in the univariate model; odds ratio 1.09, 95%CI 1.05–1.13, *p* < 0.0001 in the multivariate model), but sex was not (odds ratio 2.24, 95%CI 0.97–5.15, *p* = 0.0584 in the univariate model; odds ratio 2.06, 95%CI 0.72–5.58, *p* = 0.1758 in the multivariate model).

## Discussion

In this study, we analyzed 112 whole genome sequences of SARS-CoV-2 samples collected at Juntendo University Hospital as well as the genome data deposited in GISAID (97,458 complete sequences obtained in Japan) to investigate the pattern of mutations and the correlation of mutations with transmissibility and severity.

We found that the most predominant lineage of SARS-CoV-2 changed in each wave in Japan (Figure 1; Supplementary Tables 1, 2). It is likely that the major lineages in the later waves would be selected as a result of the advantageous transmissibility and/or immune escape potential (Korber et al., 2020; Mlcochova et al., 2021; Planas et al., 2021; Volz et al., 2021b; Wu et al., 2021).

Our sequence data showed that the viral protein with the largest number of mutations was S protein (Table 1). This reflects the important roles of the S protein in the transmission and survival of SARS-CoV-2. The amino acid substitutions in spike allow the virus to bind with greater strength to ACE2, fuse more efficiently with its target cell, and/or escape from neutralizing antibodies.

B.1.1.284 became the most widespread lineage in the second wave, replacing B.1.1, the dominant lineage in the first wave (Figure 1; Supplementary Tables 1, 2). B.1.1.284 would have higher transmissibility than B.1.1, and some newly acquired mutations in B.1.1.284 would be responsible for its enhanced transmissibility. It is implied that S: M153T, N: P151L, NSP3: S543P, NSP5: P108S, and/or NSP12: A423V in B.1.1.284 might confer the higher transmissibility of this lineage, allowing it to surpass B.1.1 (Table 2).

B.1.1.214 is considered to have elevated transmissibility in comparison with B.1.1.284 because B.1.1.214 exceeded B.1.1.284 in infections and became the major lineage in the third wave (Figure 1; Supplementary Tables 1, 2). However, B.1.1.214 has only D614G in the S protein and has fewer spike mutations than B.1.1.284 and B.1.1, which have lower transmissibility than B.1.1.284 (Table 2). These results suggest that the enhanced transmissibility of B.1.1.214 is due to amino acid changes in the viral protein rather than spike. The specific mutations of B.1.1.214 outside S protein were N: M234I, NSP14: P43L, and NSP16: R287I. It is speculated that these amino acid substitutions increase the efficiency of viral RNA replication and contribute to the wider spread of SARS-CoV-2.

Regarding the severity of infected patients’ symptoms, only patients with B.1.1.214 exhibited significantly lower severity than those with B.1.1.284 (Tables 3, 4). The absence of mutations other than D614G in S protein may be associated with lower severity of B.1.1.214. In other words, the acquisition of S protein mutations such as L54F in B.1.1 and M153T in B.1.1.284 may increase the severity of COVID-19.

R.1, which was the second most dominant lineage in the fourth wave, harbored E484K in the S protein. It is expected that E484K results in a stronger interaction between the S protein and ACE2 due to a charge switch and conformational changes (Nelson et al., 2021). Furthermore, previous reports showed that an E484K mutation reduces the neutralizing activity of convalescent and mRNA vaccine-elicited sera/plasma against SARS-CoV-2 (Cavanaugh et al., 2021; Hacisuleyman et al., 2021; Nonaka et al., 2021; Wang et al., 2021). In addition, a W152L mutation in the N-terminal domain potentially allows for immune escape (Chi et al., 2020).

The Alpha strains, including B.1.1.7 and Q.1, were most frequently detected in the fourth wave (Figure 1; Supplementary Tables 1, 2). These results indicate that the Alpha strains are more transmissible than the previously dominant strains, B.1.1.214 and R.1. Our finding is consistent with a previous report that showed an increased reproduction number of the Alpha strain (Davies et al., 2021a; Leung et al., 2021; Volz et al., 2021b). The spike of the Alpha strain has N501Y, which plays an important role in increasing affinity of S protein to ACE2 (Ali et al., 2021; Laffeber et al., 2021; Tian et al., 2021). Moreover, H69del and V70del were reported to increase infectivity through efficient incorporation of cleaved spike into virions (Meng et al., 2021).

Spike mutations N501Y, H69del, and V70del may increase severity and transmissibility, but Alpha variants showed no significant difference in severity from the reference lineage, B.1.1.284, in this study (Tables 3, 4). Our results were similar to a previous paper that did not show a significant association of the Alpha strain with higher disease severity (Davies et al., 2021a). However, it was inconsistent with a report indicating an increased mortality of B.1.1.7-infected patients (Davies et al., 2021b). The reason why the Alpha variant did not show significantly higher severity than B.1.1.284 in this study may be due to the small sample size.

The Delta strain, including AY.29 and AY.29.1, replaced the Alpha strain and became the most common strain in the fifth wave (Figure 1; Supplementary Tables 1, 2). From these results, it is concluded that the Delta strain has higher transmissibility than the Alpha strain. Previous reports also showed that the Delta strain is more transmissible than the Alpha strain (Alizon et al., 2021; Campbell et al., 2021; Liu and Rocklov, 2021; Allen et al., 2022). The Delta strains that were sequenced in our laboratory had 11 amino acid changes in the S protein, including L452R, T478K, and P681R.

The L452R is situated in the receptor binding domain (RBD) and is presumed to stabilize the complex of RBD and ACE2 (Motozono et al., 2021). The L452R mutation leads to increased infectivity of the virus. In addition to infectivity, L452R has been reported to be associated with escape from neutralizing antibodies (Deng et al., 2021). L452R caused a 3–10-fold reduction of susceptibility to about one third of vaccine and convalescent plasma samples (Ferreira et al., 2021; Greaney et al., 2021).

T478K is also located in RBD, and *in silico* analysis of spike structure has predicted that T478K may alter the electrostatic surface and increase steric hindrance of the S protein (Di Giacomo et al., 2021). It is suggested that T478K could enhance the binding affinity of RBD to ACE2.

The P681R mutation is present near the furin cleavage site and affects the efficiency of the cleavage reaction. It has been reported that P681R facilitates S protein cleavage, accelerates viral fusion and cell-to-cell infection, and enhances viral pathogenicity in hamster models (Mlcochova et al., 2021; Saito et al., 2021).

It is suspected that the Delta strain causes more severe disease than the preexisting strains due to S protein mutations. However, in the present study, the severity of the Delta strain was not significantly higher than that of B.1.1.284 (Tables 3, 4). Some studies showed that the Delta strain was associated with the higher severity (Sheikh et al., 2021; Twohig et al., 2022), while others reported that the severity of the Delta strain was not significantly elevated (Gunadi et al., 2021; Taylor et al., 2021). The reason why there is no significant difference between the Delta strain and the reference lineage in our study may be that the sample size was small, and that 44.4 percent of the patients infected with the Delta strain were vaccinated at least once while those with B.1.1.284 were not vaccinated.

In addition to the small sample size, a limitation of this research is that there is no experimental data using recombinant SARS-CoV-2 with or without specific amino acid changes to confirm the effects of the mutations.

In summary, we analyzed the sequences of 112 SARS-CoV-2 genomes detected at Juntendo University Hospital and examined the correlation of the amino acid changes with the transmissibility and the severity of each strain/lineage. It is concluded that mutations in S, N, and NSPs increase transmissibility and/or severity. These mutations include S: M153T, N: P151L, NSP3: S543P, NSP5: P108S, and NSP12: A423V in B.1.1.284; S: W152L and E484K in R.1; S: H69del, V70del, and N501Y in the Alpha strain; S: L452R, T478K, and P681R in the Delta strain. Furthermore, it is suggested that the transmissibility of the virus could be enhanced by the mutations in proteins other than spike, such as N: M234I, NSP14: P43L, and NSP16: R287I in B.1.1.214. The evolution of the virus occurs because of mutations and natural selection of the variants. To address this issue, continuous monitoring of the mutations in the viral genomes and analysis of their effects will be required to develop vaccines and antiviral drugs effective against emerging SARS-CoV-2 variants (Hoque et al., 2020; Islam et al., 2020).

## Data availability statement

The datasets presented in this study can be found in online repositories. The names of the repository/repositories and accession number(s) can be found in the article/Supplementary material.

## Ethics statement

The studies involving human participants were reviewed and approved by the institutional review board at Juntendo University Hospital. Written informed consent for participation was not required for this study in accordance with the national legislation and the institutional requirements.

## Author contributions

YT and NY conceived the study. YT, MH, YM, HM, SH, TM, KTa, and TN coordinated collection and processing the clinical samples. KTs, YH, MW, and SM processed samples. KTs and YH performed RNA isolation and RT-PCR. KTs, YH, MW, SM, and YT performed NGS experiments. KTs, YH, YT, and NY performed sequencing data analysis. NY performed phylogenetic analysis. KTs, YH, YT, NY, and SN contributed to data analysis. YT, SN, and NY prepared and wrote the manuscript. All authors contributed to the article and approved the submitted version.

## Funding

This research was partially supported by the Japan Agency for Medical Research and Development (AMED) under Grant Number JP20fk0108472 to TN and JP20fk0108505 to NY. This work was also supported in part by Research and Study Project of Tokai University to NY, and 2021–2022 Tokai University School of Medicine Project Research to NY.

## Conflict of interest

The authors declare that the research was conducted in the absence of any commercial or financial relationships that could be construed as a potential conflict of interest.

## Publisher’s note

All claims expressed in this article are solely those of the authors and do not necessarily represent those of their affiliated organizations, or those of the publisher, the editors and the reviewers. Any product that may be evaluated in this article, or claim that may be made by its manufacturer, is not guaranteed or endorsed by the publisher.

## Footnotes

^1^ https://covid19.who.int/

^2^ https://www.mhlw.go.jp/stf/covid-19/open-data.html

## Supplementary material

The Supplementary materials for this article can be found online at: https://www.frontiersin.org/articles/10.3389/fmicb.2022.912061/full#supplementary-material
