# Supplementary material for: Molecular characterization of SARS-CoV-2 detected in Tokyo, Japan during five waves: Identification of the amino acid substitutions associated with transmissibility and severity
Source: Front Microbiol. 2022 Jul 27;13:912061. doi: 10.3389/fmicb.2022.912061 (PMC9363898; doi:10.3389/fmicb.2022.912061)
Supplement: Supplementary file 1 [file Table_1.pdf]

Supplementary Table 1. Analysis of the lineages of SARS-CoV-2 collected at Juntendo University Hospital by whole genome sequencing

Wave	1st		2nd		3rd		4th		5th	All
Phase	Before peak	After peak	Before peak	After peak	Before peak	After peak	Before peak	After peak	Before peak	
Period	Mar.1, 2020- Apr.11, 2020	Apr.12, 2020- May31, 2020	Jun.1, 2020- Aug.7, 2020	Aug.8, 2020- Oct.5, 2020	Oct.6, 2020- Jan.8, 2021	Jan.9, 2021- Feb.18, 2021	Feb.19, 2021- May3, 2021	May4, 2021- Jun.24, 2021	Jun.25, 2021- Jul.31, 2021	Mar.1, 2020- Jul.31, 2021
B.1.1.285	2									2
B.1.1.48	1									1
B.1.1		4	1				1			6
B.1.1.284			6	4		1				11
B.1.1.214				3	8	4	1			16
B.1.346						2				2
B.1.1.7							14	22	8	44
Q.1								1		1
R.1							9	1		10
B.1.525								1		1
AY.29								2	14	16
AY.29.1									2	2
Total	3	4	7	7	8	7	25	27	24	112

The data presented in the study are deposited in the GISAID repository (www.gisaid.org). The accession numbers are as follows: EPI_ISL_2671835 - EPI_ISL_2671848, EPI_ISL_2671890 - EPI_ISL_2671899, EPI_ISL_2960320 - EPI_ISL_2960339, EPI_ISL_2964598 - EPI_ISL_2964624, EPI_ISL_3232536 - EPI_ISL_3232551, EPI_ISL_3908498 - EPI_ISL_3908512, EPI_ISL_4197798, EPI_ISL_5323929, EPI_ISL_5323930.2, EPI_ISL_5352023, EPI_ISL_5352027, EPI_ISL_5352033, EPI_ISL_5352037, EPI_ISL_5352043, EPI_ISL_5352049, and EPI_ISL_5352053

Supplementary Table 2. Analysis of SARS-CoV-2 lineages identified in Japan

Wave	1st		2nd		3rd		4th		5th	All
Phase	Before peak	After peak	Before peak	After peak	Before peak	After peak	Before peak	After peak	Before peak	
Period	Mar.1, 2020-Apr.11, 2020	Apr.12, 2020-May31, 2020	Jun.1, 2020-Aug.7, 2020	Aug.8, 2020-Oct.5, 2020	Oct.6, 2020-Jan.8, 2021	Jan.9, 2021-Feb.18, 2021	Feb.19, 2021-May3, 2021	May4, 2021-Jun.24, 2021	Jun.25, 2021-Jul.31, 2021	Mar.1, 2020-Jul.31, 2021
A	111	2	2	0	0	0	0	0	0	115
A.16	53	0	0	0	0	0	0	0	0	53
B	6	0	0	0	0	0	0	0	0	6
B.1	147	28	1	8	8	16	10	0	0	218
B.12	100	18	0	1	0	0	0	0	0	119
B.1.1	1331	1485	149	19	242	176	84	2	0	3488
B.1.1.48	95	245	1	0	0	0	0	0	0	341
B.1.1.285	73	36	0	0	0	0	0	0	0	109
B.1.1.283	26	16	3	0	0	0	0	0	0	45
B.1.1.465	0	64	0	0	0	0	0	0	0	64
B.1.1.482	0	27	0	0	0	0	0	0	0	27
B.1.1.284	0	13	3255	2708	2073	794	84	3	0	8930
B.1.1.214	2	1	564	1372	8043	4911	1610	27	1	16531
B.1.1.222	0	2	0	0	0	1	0	0	0	3
B.1.36	0	0	0	1	21	7	0	0	0	29
B.1.346	0	1	0	1	64	69	106	3	0	244
R.1	0	0	0	0	79	530	5331	1072	20	7032
B.1.1.7	0	7	0	0	15	184	13619	20358	10493	44676
Q.1	0	0	0	0	0	0	6	5	0	11
P.1	0	0	0	0	0	3	8	0	0	11
B.1.1.220	0	0	0	0	0	0	68	19	0	87
B.1.525	0	0	0	0	0	0	0	1	0	1
B.1.617.2	0	0	0	0	0	0	6	186	12	204
AY.29	0	0	0	0	0	2	0	368	13290	13660
AY.29.1	0	0	0	0	0	0	0	0	504	504
AY.1	0	0	0	0	0	0	0	44	1	45
AY.23	0	0	0	0	0	0	0	7	83	90
AY.24	0	0	0	0	0	0	0	15	70	85
AY.26	0	0	0	0	0	0	0	0	3	3
Others	58	32	43	4	35	50	217	108	180	727
Total	2002	1977	4018	4114	10580	6743	21149	22218	24657	97458
